# Population Structure Induces a Symmetry Breaking Favoring the Emergence of Cooperation

**DOI:** 10.1371/journal.pcbi.1000596

**Published:** 2009-12-11

**Authors:** Jorge M. Pacheco, Flávio L. Pinheiro, Francisco C. Santos

**Affiliations:** 1 Departamento de Matemática da Universidade do Minho, Campus de Gualtar, Braga, Portugal; 2 ATP-group, CFTC, Complexo Interdisciplinar, Lisboa, Portugal; 3 MLG and IRIDIA, Computer and Decision Engineering Department, Université Libre de Bruxelles, Brussels, Belgium; 4 CENTRIA, Universidade Nova de Lisboa, Quinta da Torre, Caparica, Portugal; 5 GADGET, Lisboa, Portugal; Hungarian Academy of Sciences, Hungary

## Abstract

The evolution of cooperation described in terms of simple two-person interactions has received considerable attention in recent years, where several key results were obtained. Among those, it is now well established that the web of social interaction networks promotes the emergence of cooperation when modeled in terms of symmetric two-person games. Up until now, however, the impacts of the heterogeneity of social interactions into the emergence of cooperation have not been fully explored, as other aspects remain to be investigated. Here we carry out a study employing the simplest example of a prisoner's dilemma game in which the benefits collected by the participants may be proportional to the costs expended. We show that the heterogeneous nature of the social network naturally induces a symmetry breaking of the game, as contributions made by cooperators may become contingent on the social context in which the individual is embedded. A new, numerical, mean-field analysis reveals that prisoner's dilemmas on networks no longer constitute a defector dominance dilemma—instead, individuals engage effectively in a general coordination game. We find that the symmetry breaking induced by population structure profoundly affects the evolutionary dynamics of cooperation, dramatically enhancing the feasibility of cooperators: cooperation blooms when each cooperator contributes the same cost, equally shared among the plethora of games in which she participates. This work provides clear evidence that, while individual rational reasoning may hinder cooperative actions, the intricate nature of social interactions may effectively transform a local dilemma of cooperation into a global coordination problem.

## Author Summary

Humans contribute to a broad range of cooperative endeavors. In many of them, the amount or effort contributed often depends on the social context of each individual. Recent evidence has shown how modern societies are grounded in complex and heterogeneous networks of exchange and cooperation, in which some individuals play radically different roles and/or interact more than others. We show that such social heterogeneity drastically affects the behavioral dynamics and promotes cooperative behavior, whenever the social dilemma perceived by each individual is contingent on her/his social context. The multiplicity of roles and contributions induced by realistic population structures is shown to transform an initial defection dominance dilemma into a coordination challenge or even a cooperator dominance game. While locally defection may seem inescapable, globally there is an emergent new dilemma in which cooperation often prevails, illustrating how collective cooperative action may emerge from myopic individual selfishness.

## Introduction

Portuguese is no exception: Like any other language, it has many proverbs and popular sayings. One of them states something like: *I have already contributed to that charity* [1], concerning originally situations in which individuals are faced with the decision of offering (or not) a contribution to a common venture, the expression above meaning “*no*”. Interestingly, the amount given is never stated. It turns out that, quite often, we are confronted with situations in which the act of giving is more important than the amount given. Let us keep with a charity event, in which some celebrities are invited to participate. Typically their appearance is given maximal audience, and they are shown contributing a seemingly large amount of money to the charity's cause. This offer is aimed at stimulating the contribution of many to the same charity, and indeed this mechanism of “celebrity participation in charities” is common, and presumably effective. But what is the relevance of the amount contributed by the celebrity? It is certainly impressive to many, despite being, most likely, a small contribution, both in face of the celebrity's wealth and also in what concerns the overall amount accumulated. But it does induce, hopefully, a large number of (much smaller) contributions from anonymous (non-celebrities, the overwhelming majority) charity participants, who feel compelled to contribute given the fact that their role model (the celebrity) contributed. In other words, the majority copies (imitates) the act of giving, but certainly not the amount given.

Nowadays, web-signed petitions are also examples of collective decisions which, often, benefit from the fact that some well-known people adhere to the petition's cause. Besides those who are fully aware and agree with the cause, there are also those who sign the petition simply because they admire someone who has signed the petition, again copying the attitude. Many other examples from real life could be provided along similar lines, from trivia, to fads, to stock markets, to Humanitarian causes up to the salvation of planet Earth [2]–[4]. From a theoretical perspective, many of these situations provide beautiful examples of public goods games [5],[6] (**PGG**) which are often hard to dissociate from reputation building, social norms and moral principles [7]–[11]. This intricate interplay reflects the many-body nature and multi-level complexity of the interactions among the “social atoms” [12].

The simplest **PGG** involves two persons. Both have the opportunity to contribute a cost *c* to a common pool. A Cooperator (***C***) is one who contributes; otherwise she is a Defector (***D***). The total amount is multiplied by an enhancement factor *F* and equally shared between the two participants. Hence, player *i* (*i* = 1, 2) using strategy *s_i_* (*s_i_* = 1 if ***C***, 0 if ***D***) gets a payoff 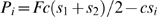 from this game, leading to the following payoff matrix
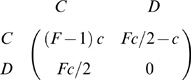
(1)


For 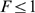 ***D***s dominate unconditionally. For *F = 2* no strategy is favored in well mixed populations (neutral drift); yet, for 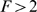, it is better to play ***C*** despite the fact that, in a mixed pair, a ***D*** collects a higher payoff than a ***C***. For 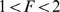 the game is an example of the famous symmetric one-shot two-person prisoner's dilemma [13], on which many central results have been obtained over the years, in particular in the context of evolutionary game theory [14],[15]: In 1992 [16] it has been explicitly shown that population structure matters, despite its importance being recognized already by Darwin, albeit in the form of Group selection [17],[18]. It clearly makes a difference whether everybody is equally likely to interact with anybody else in the population or not (see also [19]). In 2004 we learnt that evolutionary game theory in finite populations may behave very differently from that on infinite populations [20], even in the absence of any population structure, Evolutionarily Stable Strategies (ESS) becoming population size dependent. In 2005 we learnt that heterogeneous population structures play an important role in the evolution of cooperation under the prisoner's and other social dilemmas [21],[22], a result which spawned a number of investigations [23]–[29] (see also Szabó and Fáth for a recent review [30]). In 2006 a mathematical condition was obtained for ***C***s to become advantageous on populations structured along the links of homogeneous networks [31], subsequently confirmed making use of inclusive fitness methods [32] for a limited subset of game payoff matrices. This result, valid in the limit of weak selection, has also unraveled an important feature of evolutionary game theoretical studies: The outcome of cooperation depends on the evolutionary dynamics adopted, dictating how individual strategy evolves from generation to generation. Furthermore, evolutionary game dynamics on populations structured along multiple networks has been explored [33],[34], as well as the mechanisms which favor cooperation under adaptive population structures have been identified, both for non-repeated [35]–[43] and repeated games [44],[45]. These results consubstantiate and keep stimulating an enormous amount of research work.

Common to all these studies are the settings underlying the social dilemma: in the conventional view, every ***C*** pays a fixed cost *c* per game, providing the same benefit *b* to the partner. However, if what matters is the act of giving and not the amount given, then there is no reason to assume that everybody contributes the same cost *c* to each game. Depending on the amount of each individual contribution, the overall result of the evolutionary dynamics may change. The two person game introduced above provides not only the ideal ground to introduce such a diversity of contributions, but also an intuitive coupling between game dynamics and social embedding: The first (second) individual contributes a cost *c_1_* (*c_2_*) if playing ***C*** and nothing otherwise. Hence, player *i* (*i = 1, 2*) now gets the following payoff from this game:
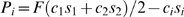
(2)reflecting the symmetry breaking induced by possibly different contributions from different cooperating individuals. This poses a natural question: Who will acquire an evolutionary edge under these conditions?

Often the amount that each individual contributes is correlated with the social context she is actually embedded in [28],[46],[47]. Modern communities are grounded in complex social networks of investment and cooperation, in which some individuals play radically different roles and interact more and more often than others. Empirical studies have demonstrated that social networks share both small-world properties and heterogeneous distribution of connectivities [48]–[50]. In such heterogeneous communities, where different individuals may be embedded in very different social environments, it is indeed hard to imagine that every ***C*** will always provide the same amount in every game interaction, hence reducing the problem to the standard two-person prisoner's dilemma studied so far. In the context of N-person games played in prototypical social networks, it has been found that the diversity of contributions greatly favors cooperation [28]. However, and similar to the relation between two-body and many-body interactions in the Physical Sciences, N-person public goods games have an intrinsic complexity which cannot be anticipated from two-person games: In the words of late William Hamilton, “*The theory of many person games may seem to stand to that of two-person games in the relation of sea-sickness to a headache*” [51].

Here, and besides the conventional scenario in which every ***C*** contributes the same cost *c* to each game she participates, we shall also explore the limit in which every ***C*** contributes the same overall amount *c*. However, this amount is *shared* between all games she participates, which are defined by the social network in which the players are embedded. For instance, *c* may be interpreted as the availability or the amount of resources each individual has to dedicate to all her commitments. Hence, the contribution to each game will depend now on the social context (number of partners) of each ***C***, and heterogeneity will foster a symmetry breaking of pair-wise interactions, as two individuals may contribute different amounts to the same game. In this sense, cooperation will be identified with the act of giving and no longer with the amount given.

## Results

**Figure 1:**
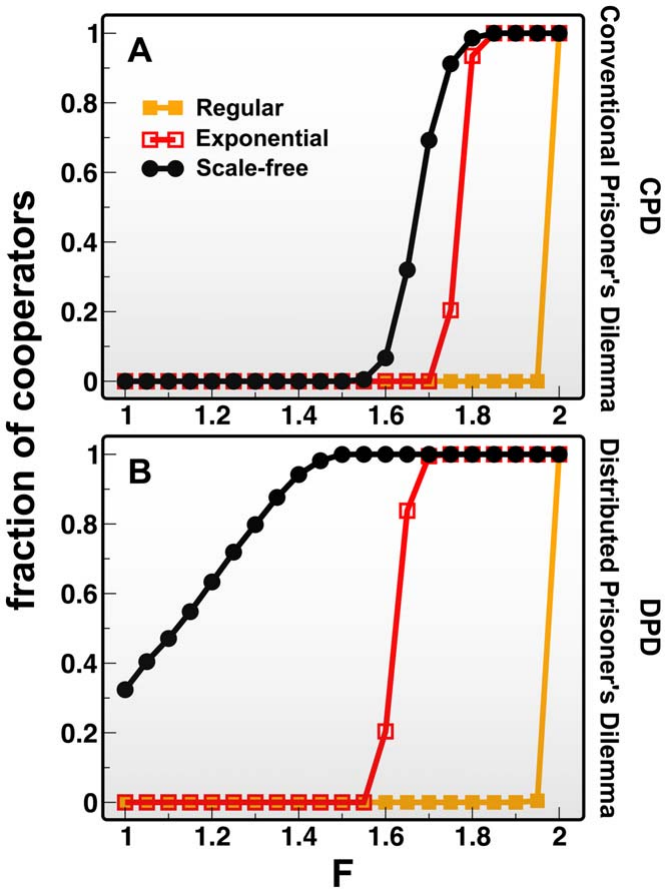
Fraction of Cooperators as a function of the enhancement factor *F*. Upper panel: Under CPD Cooperation is able to dominate on Scale-free networks (lines and circles), unlike what happens on regular structures (lines and filled squares). On exponential networks, intermediate levels of cooperation emerge, as a result of the heterogeneity of such topologies. Lower panel: Under DPD the advantage of Cs is dramatically enhanced when the same cost is evenly shared among each one's neighbors. The results were obtained for networks of *10^3^* nodes and an average degree *z = 4*. As expected, abandoning the well-mixed regime leads to a break-up of neutrality for *F = 2*.

Figure 1 shows the final fraction of ***C***s for different classes of population structures and different contribution paradigms. At each time-step, every individual engages in a 2-person **PGG** with each of her neighbors. The accumulated payoff resultant from all interactions is associated with reproductive fitness or social success, which determines the behavior in the next generation [15]. We adopt the so-called pairwise comparison rule [52]–[54] for the social learning dynamics: Each individual copies the behavior of a randomly chosen neighbor with a probability which increases with the fitness difference (see Methods for details).

Figure 1a shows the outcome of evolving the conventional 2-person **PD** (*1<F<2*), in which case each player contributes a fixed amount *c* to each game she participates. Different population structures are considered, one associated with a (homogeneous) regular network (**REG**), the other with a (strongly heterogeneous) scale-free network (**SF**). Real social networks fall somewhere between these limits [55], and hence we also investigate a third class of population structure, represented by an exponential network (**EXP**), exhibiting a level of heterogeneity intermediate between the previous two.

The existence of a minority of highly connected individuals in **SF** networks (line and circles) allows the population to preserve high cooperative standards, while on homogeneous networks (line and filled squares) ***D***s dominate for the entire range of parameters [21],[22], as a result of the pairwise comparison rule adopted [56]. Heterogeneous networks thus pave the way for the emergence of cooperation. Highly connected individuals (i.e. *hubs*) work as catalysers of cooperative behavior, as their large number of interactions allows them to accumulate a high fitness. This, in turn, leads them to act as role models for a large number of social ties. To the extent that hubs are ***C***s, they influence the vast majority of the population to follow their behavior [23]. Clearly, this feature has a stronger impact on **SF** networks than on **EXP** networks, the difference between these two types of networks stemming from the presence or absence, respectively, of the preferential attachment mechanism.

The results in Figure 1a are based on the assumption that each ***C*** contributes the same cost *c* to each game she plays – which we denote by *conventional prisoner's dilemma* (**CPD**). This assumption is relaxed in Figure 1b where ***C***s now equally distribute the same cost *c* among all games they play – the regime we denote by *distributed prisoner's dilemma* (**DPD**). Figure 1b shows what happens in this limit. While on homogeneous networks the fate of cooperation is the same as before − it amounts to rescaling of the intensity of selection − heterogeneity in the amount contributed by each individual to each game creates a remarkable boost in the final number of ***C***s for the entire range of *F*, which increases with increasing heterogeneity of the underlying network. Comparison with the results of Figure 1a shows that under **DPD** preferential attachment plays a prominent role, since it constitutes the network wiring mechanism distinguishing **EXP** networks from **SF** networks. Changing from **CPD** to **DPD** induces moderate boosts in the equilibrium fraction of ***C***s on **EXP** networks, but a spectacular boost of cooperation on **SF** networks: Hubs become extremely influential under **DPD**.

**Figure 2:**
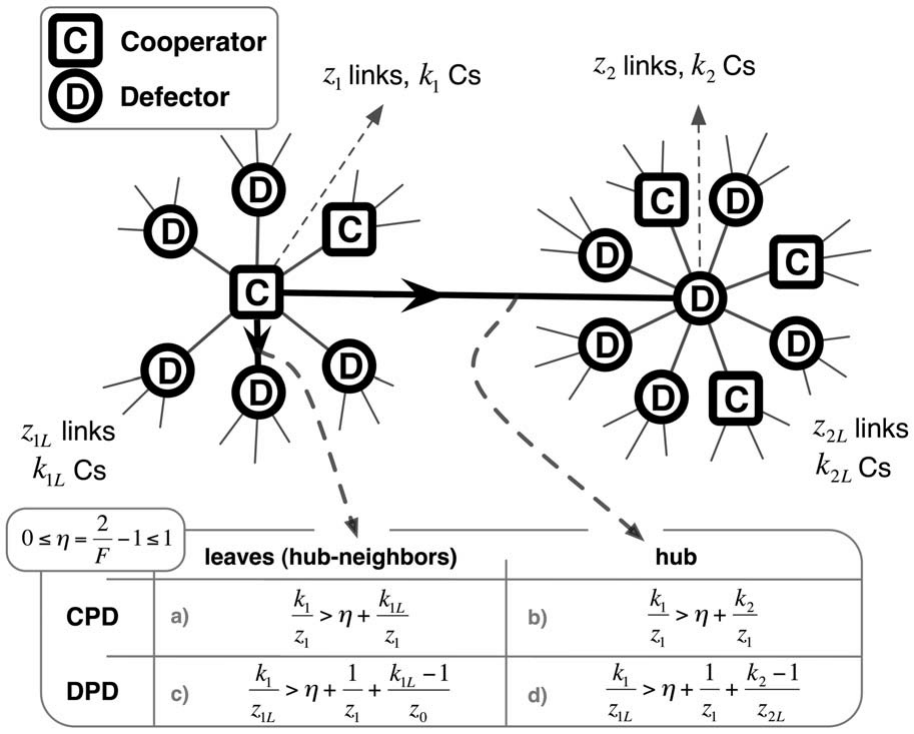
Invasion conditions for a hub-cooperator. From the definitions of the parameters in the figure one obtains that DPD leads systematically to less stringent conditions for invasion of the *C* (squares) occupying the left hub, explaining the increased success of Cs under DPD. On general heterogeneous populations with average connectivity *z_0_*, conditions a) and b), as well as c) and d), show that it is easier to invade a *D* (circles) on a leaf than in the center of another hub. This invasion creates a positive feedback resulting from cooperative “leaves” surrounding the left hub (*k_1_ - k_2_* increases) allowing a subsequent invasion of the right hub.

In order to understand the mechanism underlying the population-wide boost of cooperation obtained, we consider a prototypical element of a heterogeneous network (similarly to what has been done in [28],[30],[31]) as shown in Figure 2, and investigate the *microscopic* balance determining individual change. In particular, we investigate under which conditions the central ***C*** on the left – a stereotypical *hub* –becomes advantageous, that is, accumulates a higher fitness than any of her neighbors (see Figure 2). We consider a ***C*** *-hub* with z_1_ links (*k_1_* of which are ***C***s, left in Figure 2) and a ***D*** *-hub* with z_2_ links (*k_2_* of which are ***C***s, right in Figure 2). We assume, for simplicity, that all neighbors of the ***C*** hub have z_1L_ links each (*k_1L_* of which are ***C***s), whereas all neighbors of the ***D*** hub have z_2L_ links (*k_2L_* of which are ***C***s). The remaining nodes have z_0_ links, where z_0_ stands, e.g., for the average connectivity of the population. We implicitly assume that the neighbors of the hubs have smaller connectivities, and consequently we call them *leaves*.

The conditions are explicitly provided in Figure 2 for both **DPD** and **CPD**. In both paradigms, for the ***C*** *-hub* to invade the ***D*** *-hub* (or any of her ***D*** *-leaf* neighbors) depends crucially on the difference between the number *k_1_* of ***C*** *-neighbors* of the ***C*** *-hub* and the number *k_2_* (*k_1L_*) of ***C*** *-neighbors* of the ***D*** *-hub* (***D*** *-leaf*). In both **DPD** and **CPD** the invasion threshold is always smaller for leaf invasion compared to hub invasion. Furthermore, the threshold for invasion is also smaller under **DPD** compared to **CPD**. Finally, as one would expect, all thresholds coincide when networks are homogeneous, the threshold conditions making it harder for invasion to occur in these networks. As a result, on heterogeneous networks, the conditions which render a ***C*** *-hub* advantageous with respect to a ***D*** *-hub* are more stringent than those associated with invasion of a neighbor ***D*** *-leaf*, which leads to an invasion pattern in which leaves are invaded before hubs [23]. Furthermore, one should not overlook that successful ***D***s tend to place other ***D***s in their neighborhood [23] which acts as a negative feedback mechanism reducing their fitness in time. On the contrary, successful ***C***s see their fitness increase in time, as more ***C***s join their neighborhood, reinforcing their fitness.

**Figure 3:**
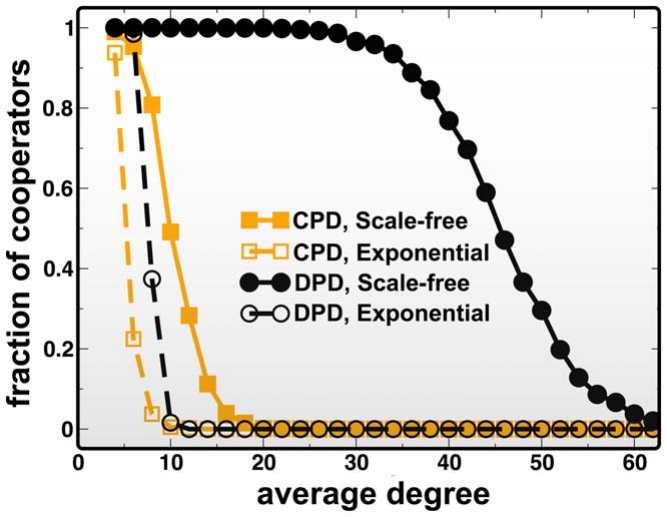
Fraction of Cooperators as a function of the average degree *z* of the social network. Cooperation is able to dominate on sparse networks. Yet, only under **DPD**, combined with high levels of heterogeneity of Scale-free networks, one observes the maintenance of cooperative behavior in highly connected populations. The results were obtained for networks of *10^3^* nodes and *F = 1.8*.

The impact of the **DPD** paradigm, however, is most dramatic if one takes into consideration that the condition for the ***C*** *-hub* to become advantageous becomes less stringent the larger her connectivity. On the contrary, under the **CPD** paradigm, the cost of cooperation plays a major role in the overall fitness of the ***C*** *-hub*, which means that the larger her connectivity, the harder it will be for the ***C*** *-hub* to become advantageous with respect to any of her ***D*** *-neighbors*. Finally, the threshold conditions in Figure 2 also show that under **DPD** the range of game interaction is enlarged, as second neighbors of a hub also play a role in defining the invasion thresholds, unlike what happens under **CPC**. The insights provided by the prototypical configuration in Figure 2 become more explicit if one computes the outcome of cooperation in ***SF*** networks for social networks with different average connectivities and both contributive schemes (Figure 3). As the average degree (z) becomes sizable cooperation will inevitably collapse [21],[23], but while cooperation can hardly resist for z *>10* in the case of **CPD**, under **DPD** ***C***s survive for values of z roughly four times larger. This is of particular importance given that social networks often exhibit high average connectivity values (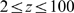) [48]: Cooperation prevails under a **DPD** contributive system, even on non-sparse static network structures. For intermediate regimes of heterogeneity (**EXP** networks), under **DPD** cooperation is also sustained up to higher values of z, but to a lesser extent: Once more, the impact of large hubs resulting from the preferential attachment mechanism underlying **SF** networks plays an impressive role under **DPD**.

**Figure 4:**
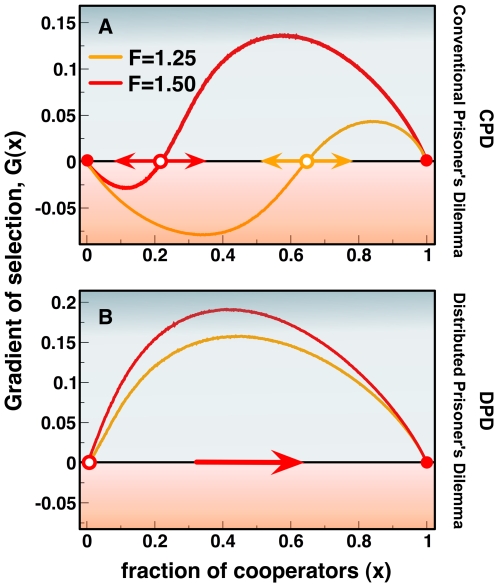
Gradients of selection *G(x)*. a) Under the CPD paradigm, Scale-free networks lead to the appearance of an unstable equilibrium x* (open circles) and a scenario characteristic of a coordination game, paving the way for cooperator dominance for frequencies above x*. b) Under DPD, *G(x)* becomes positive for (almost) all values of *x* (x*<0.004 for *F = 1.50* and x*<0.006 for *F = 1.25*), leading to a scenario characteristic of a Harmony game, where cooperators dominate unconditionally. In both panels the networks employed had *500* nodes and an average degree *z = 4*, whereas *β* = 10.0.

The previous analysis allowed us to understand in which way heterogeneous networks, by inducing a symmetry breaking into the game dynamics, may favor cooperation. Furthermore, Figures 1 and 3 show how this indeed happens when one starts from initial conditions in which ***C***s occupy the nodes of a network with 50% probability. This approach, which is recurrent in numerical studies of evolutionary game dynamics, contrasts with the more conventional mean field analysis on which evolutionary game theory is rooted. There, the fact that all ***C***s and ***D***s in an infinite population have the same fitness, leads to a simple replicator equation in which the rate of change of ***C***s is proportional to a Gradient of selection *G(x)*, the interior roots of which dictate possible coexistence or coordination equilibria [14]. Here we shall define the finite population analog of *G(x)*, valid for any population size and structure (see Methods). In doing so we overlook the microscopic details of the competition and self-organization of Cs and Ds, but we gain an overview of the game dynamics in a *mean-field perspective*. *G* becomes positive whenever cooperation is favored by evolution and negative otherwise. Whenever *G = 0*, selection becomes neutral and evolution proceeds by random drift. Naturally, *G* will depend implicitly on the population structure, on the fraction *x* of ***C***s and also on how these ***C***s are spread in the network. In Figure 4 we plot *G(x)* as a function of *x*, for different values of *F* and different game paradigms (**CPD** and **DPD**). Each configuration, here characterized by *x*, was generated assuming that each ***C*** (***D***) has, at least, one ***C*** (***D***) in her neighborhood, replicating the conditions observed in all numerical simulations. This is an important point, as strategy assortation constitutes a characteristic feature of evolutionary game dynamics in structured populations.

Figure 4 shows that, unlike what happens on homogeneous networks, where ***D***s are always advantageous (not shown), ***SF*** networks effectively transform a prisoner's dilemma into a different game. Figure 4a indicates that, in the case of **CPD**, introducing diversity in roles and positions in the social network effectively leads to a *coordination game* [57],[58], characterized (in an infinite, well-mixed population) by a critical fraction x* above which ***C***s are always advantageous (*G<0* for x<x* and *G>0* for x>x*). This result provides a powerful qualitative rationale for many results obtained previously on heterogeneous networks under strong selection [21],[22],[28] in which degree heterogeneity is shown to induce cooperative behavior, inasmuch as the initial fraction of ***C***s is sufficient to overcome the coordination threshold. Moreover, Figure 4b shows that changing the contributive scheme from **CPD** to **DPD** in ***SF*** population structures acts to change a prisoner's dilemma effectively into a Harmony game where ***C***s become advantageous irrespectively of the fraction of ***C***s (x*≈*0*).

## Discussion

The present study puts in evidence the impact of breaking the symmetry of cooperative contributions to the same game. On strongly heterogeneous networks, the results of Figures 1b and 3 provide an impressive account of the impact of this diversity of contributions. Overall, our results strongly suggest that whenever the act of cooperation is associated to the act of contributing, and not to the amount contributed, cooperation blooms inasmuch as the structure of the social web is heterogeneous, leading individuals to play diverse roles. The multiplicity of roles and contributions induced by the social structure effectively transforms a *local* cooperative dilemma into a *global* coordination game [57]. The latter embodies an exemplary representation of the social contracts [57] found in several instances of animal [59],[60] and human [61],[62] collective dilemmas. This work provides additional evidence that, while locally, cooperation can be understood as a prisoner's dilemma, globally, the possibilities opened by the intricate nature of collective dynamics of cooperation [63] often lead to a dynamical portrait that is effectively described by a coordination dilemma instead of a defection dominance dilemma [57].

## Methods

Each individual is assigned to a node of a network, whereas interactions are represented by links between nodes. In each generation, all pairs of individuals directly connected, engage in a single round of the game. As usual, the accumulated payoff from all interactions emulates the individual *fitness* (*f_i_*) or *social success* and the most successful individuals will tend to be imitated by their neighbors. Such behavioral evolution is implemented using the pairwise comparison rule [52],[54]: at each time step an individual *x* will adopt the strategy of a randomly chosen neighbor *y* with a probability given by the ubiquitous Fermi distribution 
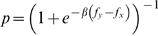
 from statistical physics [52],[54], in which *β*, the inverse temperature in Physics, translates here into noise associated with errors in decision making. For high values of *β* we obtain the imitation dynamics commonly used in cultural evolution studies whereas for *β*≪1 evolution proceeds by random drift. The strong selection regime that we adopt here (*β* = 10.0) enhances both the influence of the payoff values in the individual fitness and the role played by the social network. It is noteworthy that a detailed study of the impact of *β* on game dynamics on heterogeneous networks is still lacking, unlike what happens on homogeneous networks [52],[54],[64]. The results in Figures 1 and 3 were obtained for populations of N* = 10^3^* individuals starting with 50% of ***C***s randomly distributed on the network. In all cases we used the value *c = 1* for the cost of cooperation. The scale-free networks were generated using a direct implementation of the Barabási-Albert (***BA***) model, based on growth and preferential attachment [65], whereas exponential networks were generated replacing the preferential attachment by uniform attachment in the previous model [49]. Different mechanisms could be used [38], [42], [48], [66]–[68] to generate ***SF*** degree distributions portraying features not present in the ***BA*** model. In general, however, ***SF*** networks lead to evolutionary dynamical behaviors which are similar to those observed in ***BA*** networks [24], [27], [42], [68]–[70], which may also depend on the way individual fitness is defined [23],[29],[71],[72]. The equilibrium fraction of ***C***s results from averaging over 2000 generations after a transient period of 10^5^ generations and each point in Figures 1 and 3 corresponds to an average over *10^3^* runs and networks. The results are independent from the updating strategy (synchronous, asynchronous), population size (N >500) and robust to the existence of a small number of mutations in each time-step. In Figure 4, gradients of selection were obtained by calculating 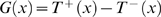, where 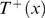 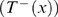 is the average frequency of transitions increasing (decreasing) the number of ***C***s for each random configuration with *x*N ***C***s. *G(x)* represents a finite population analogue (using the pairwise comparison rule [52],[54]) of the gradient of selection in infinite well-mixed populations 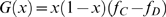 [14], where 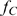 and 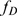 are the fitness values of ***C***s and ***D***s. Each value was obtained by averaging over *10^5^* different randomly generated configurations and networks.
